# Water soaking improves pizza bake properties of fat-free mozzarella cheese shreds

**DOI:** 10.3168/jdsc.2025-0879

**Published:** 2025-11-21

**Authors:** Suresh Sutariya, Prafulla Salunke

**Affiliations:** 1 Dairy and Food Science Department, South Dakota State University, Brookings, SD 57007; 2 IFF–Food Biosciences, New Century, KS 66031

## Abstract

Fat-free mozzarella is valued for its low-calorie, low-fat nutrition, but suffers from poor melting, excessive browning, and rubbery texture when baked, limiting its appeal. This study builds on prior findings that soaking fat-free mozzarella shreds in water improves pizza baking performance. The research evaluates the effects of soaking on frozen-thawed shreds and on leftover pizza after refrigeration and microwave reheating, using rheometer-based analysis. Results show that soaking lowers the melting temperature and enhances flowable texture, with improvements persisting after freeze-thaw cycles and reheating. Hydrating shreds thus consistently improves performance of fat-free mozzarella pizza.

## Highlights

• Nutritious fat-free mozzarella often lacks good melting and browning qualities.
• Soaking cheese shreds improves pizza melting, browning, and freeze quality.
• Bake quality persists even after shred freezing and pizza reheating.
• Rheological analysis confirms lower melting temperature as a function of soaking.
• This technique offers a healthier alternative without compromising quality.

## Graphical Abstract

**Figure:**
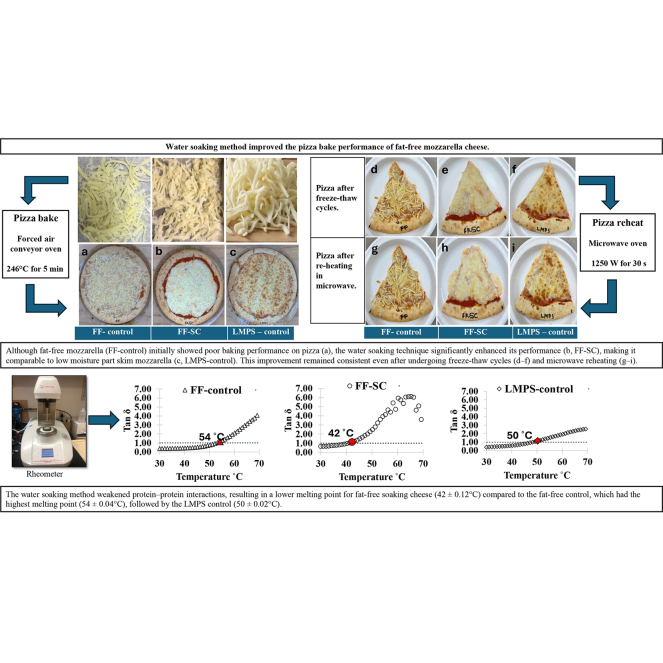
**Summary:** Fat-free mozzarella is known to have poor baking performance on pizza application. The water soaking method improved the performance of fat-free mozzarella, making it comparable to low-moisture part-skim mozzarella. This improvement is consistent after freeze-thaw cycles and microwave reheating. The water soaking method weakened protein-protein interactions, resulting in a lower melting point for fat-free soaked cheese compared with the fat-free control, which had the highest melting point, followed by the low-moisture part-skim control. Overall, this study provides valuable insights into improving the functional properties of fat-free mozzarella cheese, contributing to its broader acceptance and use in pizza applications.

Mozzarella cheese, a staple in pizza, is a fresh, unripened cheese made from buffalo or cow milk. Its stringy texture comes from the pasta filata process, where curd is heated, kneaded, and stretched, aligning protein fibers and fat globules to create a smooth, elastic, homogeneous curd. Subsequent steps include cutting, molding, and cooling to achieve structural firmness (FAO and WHO, 2006; Akhtar et al., 2023; Gislon et al., 2023). Globally, the mozzarella cheese market was valued at USD 37.5 billion in 2022 and is projected to expand to USD 67.79 billion by 2032, reflecting a compound annual growth rate of 6.80% during the forecast period (2023–2032; Singh, 2025). In the United States, ∼75% of mozzarella production is allocated for pizza manufacturing, underscoring the importance of its sensory and functional attributes in baked applications (Sattar et al., 2015).

In response to evolving consumer preferences and increasing demand for reduced-fat food products, there is growing interest in developing low-fat and fat-free mozzarella variants that emulate the sensory and functional properties of low-moisture part-skim (**LMPS**) mozzarella. Key performance indicators for mozzarella on pizza include complete melting, shred fusion, controlled browning, and the presence of free oil after baking (Jana and Tagalpallewar, 2017). Mozzarella cheese formulations with fat content lower than that of LMPS variants generally exhibit suboptimal bake performance in pizza applications. These deficiencies manifest as insufficient meltability, excessive shred identity, and heightened browning intensity (Rudan and Barbano, 1998; Wadhwani et al., 2011). Compositional parameters, specifically fat and moisture content, are pivotal in determining the functional behavior of mozzarella cheese under baking conditions (Jana and Tagalpallewar, 2017). Fat-free mozzarella poses unique challenges due to its reduced moisture-to-protein ratio and the absence of fat content. In contrast, fat-containing variants such as LMPS and whole-milk mozzarella demonstrate superior baking characteristics, notably the formation of uniformly distributed blisters ranging from 5 to 15 mm in diameter, with a desirable light- to golden-brown coloration (Dairy Pipeline, 2022). These visual and textural attributes are largely attributed to the presence of fat and moisture, which play a critical role in modulating thermal and structural dynamics during baking. Fat in mozzarella cheese serves 2 essential functional roles during baking. First, it melts and migrates to the surface of the cheese during pizza bake, forming a hydrophobic layer that mitigates moisture evaporation at cheese surface. Second, it acts as a plasticizer within the protein matrix, disrupting intermolecular protein interactions and thereby facilitating the formation of a more open and flexible network. This structural modification enhances meltability and promotes dynamic moisture redistribution. The increased porosity of the protein matrix allows for more efficient water migration from the interior to the cheese surface during baking, which helps replenish evaporated moisture. This process contributes to surface cooling and delays excessive drying, facilitating cheese melting and moderating the rate of browning, particularly in the initial stages of thermal exposure. In contrast, fat-free mozzarella exhibits a tightly packed protein structure that restricts internal water mobility. Consequently, moisture reaches the surface at a slower rate, leading to insufficient compensation for evaporative losses. This accelerates surface dehydration, causing the cheese shreds to desiccate prematurely and impeding proper melting, which results in uneven and excessive browning during baking.

Extensive research has aimed to improve the baking performance of fat-free and LMPS mozzarella cheese. Studies by Rudan and Barbano (1998) found that surface free oil helps prevent dry surface layers on cheese shreds, reducing baking defects in fat-free mozzarella. Hydrophobic coatings like cooking oil can mitigate browning and enhance meltability. Other approaches focus on increasing the moisture-to-protein ratio (Merrill et al., 1994; Fife et al., 1996), which improves hydration and reduces drying, but excessive moisture (>60%) can cause processing issues such as sticky texture and poor shredding. To address these challenges, a postshredding hydration technique soaking fat-free mozzarella shreds in cold water overnight was developed (Sutariya et al., 2022), improving baking performance by enhancing protein hydration and moisture migration. This method is cost-effective, clean-label compatible, and commercially feasible. However, previous studies mainly assessed immediate baking performance and relied on visual meltability observations, lacking quantitative analysis and not considering frozen-thawed shreds or reheated leftover pizza scenarios. To address these gaps, the current short study aims to (1) examine the effects of soaking fat-free mozzarella shreds on the baking performance of frozen-thawed cheese shreds in pizza, as well as after refrigeration storage and microwave reheating of leftover pizza; and (2) characterize the melting behavior using rheological measurements to provide a data-driven understanding of hydration effects on cheese functionality.

For the pizza bake evaluation, fat-free and LMPS shredded mozzarella cheese (Great Value brand) were procured from a commercial retailer (Walmart, New Century, KS). The method described by Sutariya et al. (2022) was followed. The cheese samples were divided into 3 groups: fat-free (**FF**) control (fat-free mozzarella shreds), fat-free soaked cheese (**FF-SC**: fat-free mozzarella shreds soaked in a 5.25 pH-adjusted, 2% salt solution), and LMPS control (LMPS mozzarella shreds with ∼48.5% moisture content). The FF-control group served as a negative control to represent the baseline limitations of fat-free mozzarella in terms of meltability, stretchability, and browning during baking. The FF-SC group was evaluated to determine the extent of improvement resulting from water soaking (hydration) treatment, and LMPS mozzarella, widely used in pizza applications, was included as a positive control to assess whether the performance of FF-SC could be enhanced to match that of conventional LMPS cheese. To optimize hydration in the FF-SC treatment, 200 g of fat-free mozzarella shreds were immersed in 100 g of a salt solution for 8 h at a refrigerated temperature of ∼5°C. The salt solution was prepared using deionized water containing 2% (wt/wt) sodium chloride, with pH adjusted to 5.25 using lactic acid (88%, Lab Alley, Austin, TX) to replicate the salt content and pH conditions of typical cheese. At the end of the soaking period, the cheese shreds had fully absorbed the salt solution. All 3 cheese samples were frozen (−18°C) for 7 d and subsequently thawed over the duration of 7 d (5°C). These frozen-thawed cheese samples were evaluated for pizza bake property evaluations.

The meltability, browning, and stretch characteristics of frozen-thawed samples of FF-control, FF-SC, and LMPS-control mozzarella were assessed using a pizza baking protocol adapted from previously validated methodologies (Rudan and Barbano, 1998; Wadhwani et al., 2011). A 12-inch thick-crust base (132 g; Alive and Kickin' Pizza Crust, Green Bay, WI) was uniformly coated with 70 g of commercial pizza sauce (Roma Food, Richmond, VA). One half of the pizza was topped with 110 g of FF-control cheese and the other half with 165 g of FF-SC cheese (comprising 110 g of cheese hydrated with 55 g of salt solution, maintaining a 2:1 ratio). Separately, the LMPS-control sample was applied to a full pizza base using 220 g of cheese. All pizzas were baked concurrently in a forced-air conveyor oven (Model PS520E, Middleby Marshall, Elgin, IL) at 246°C for 5 min. These baking parameters were selected based on recommendations from major US mozzarella manufacturers. A single baking condition was employed to isolate the effects of the hydration treatment. Baked pizza cheese images were captured using an iPhone 8 camera (Model MQ722LL/A, software version 13.6.1). Cheese stretchability was evaluated using a fork test after the baked pizza cooled to ∼77°C. A stainless-steel fork was inserted into the cheese and vertically lifted to compare stretch behavior across the 3 sample groups. Additionally, to simulate typical consumer behavior involving leftover pizza consumption, baked pizzas were stored at ∼5°C for 4 h in a cardboard pizza container. A single slice (one-sixth of the pizza) was reheated in a microwave oven (Panasonic Inverter, 1250W Genius Sensor) for 30 s at full power. Images were taken before and after reheating to document changes in browning and melting characteristics, and stretchability was reassessed using the fork test.

The rheological behavior of mozzarella cheese samples during thermal melting and subsequent cooling (resolidification) was evaluated using a modified protocol based on the method described by Rukke et al. (2018). Measurements were conducted using a modular compact rheometer (MCR302e, Anton Paar) equipped with a parallel plate geometry (PP20/S-S-SN13685) and integrated temperature control systems, including fluid circulators and a Peltier hood. Cheese samples (FF-control, FF-SC, and LMPS-control) were manually shaped into spherical forms and positioned centrally on the lower plate. They were then compressed to a uniform thickness of 2 mm between the plates. Any excess material extruded from the edges was carefully removed using a spatula, and the exposed surface was coated with mineral oil to minimize moisture loss during testing. Before measurement, samples were allowed to recover from compression for 60 s, followed by a preheating phase at 30°C for 2 min to ensure thermal equilibrium. A temperature sweep was performed from 30°C to 70°C and subsequently back to 30°C within the linear viscoelastic region, using a constant shear strain of 0.5% and a frequency of 1 Hz. The temperature increased by 1°C every 20 s. Rheological parameters including temperature, loss factor (**Tan δ**), storage modulus (**G′**), and loss modulus (**G″**) were recorded and plotted to compare the melting and resolidification profiles of FF-control, FF-SC, and LMPS-control mozzarella samples.

Our earlier research (Sutariya et al., 2022) evaluated the pizza baking performance of soaked FF-SC compared with FF-control (negative control) and a LMPS-control (positive control). The FF-control cheese exhibited poor baking characteristics, including minimal melting, pronounced shred identity, and no stretchability. In contrast, the FF-SC sample demonstrated improved melting, stretching, and browning, closely resembling the performance of the LMPS-control cheese.

Widespread use of frozen-thawed cheese shreds by pizza chains and ready-to-bake frozen pizzas represent a significant segment of the US pizza market. To reflect this reality, we assessed the baking performance of frozen-thawed versions of FF-control, FF-SC, and LMPS-control cheese shreds. The freeze-thaw cycle did not noticeably alter the meltability or browning of any of the cheese types (Figure 1b) compared with their fresh, unfrozen counterparts (Figure 1a). However, the FF-SC and LMPS-control samples appeared softer and exhibited fewer cheese strands after thawing (Figure 1b), relative to the unfrozen samples (Figure 1a). This change may be due to structural disruptions in the protein matrix, likely caused by partial dehydration and ice recrystallization during freezing and thawing (Digvijay et al., 2025). Based on visual assessment, the slight increase in softness and reduction in strand formation in the frozen-thawed samples are unlikely to negatively influence consumer perception, though further sensory evaluation is needed to confirm this. The FF-control cheese also felt softer during fork testing after thawing but still failed to exhibit any stretch, consistent with its performance before freezing (Figure 1a and b).

**Figure 1:**
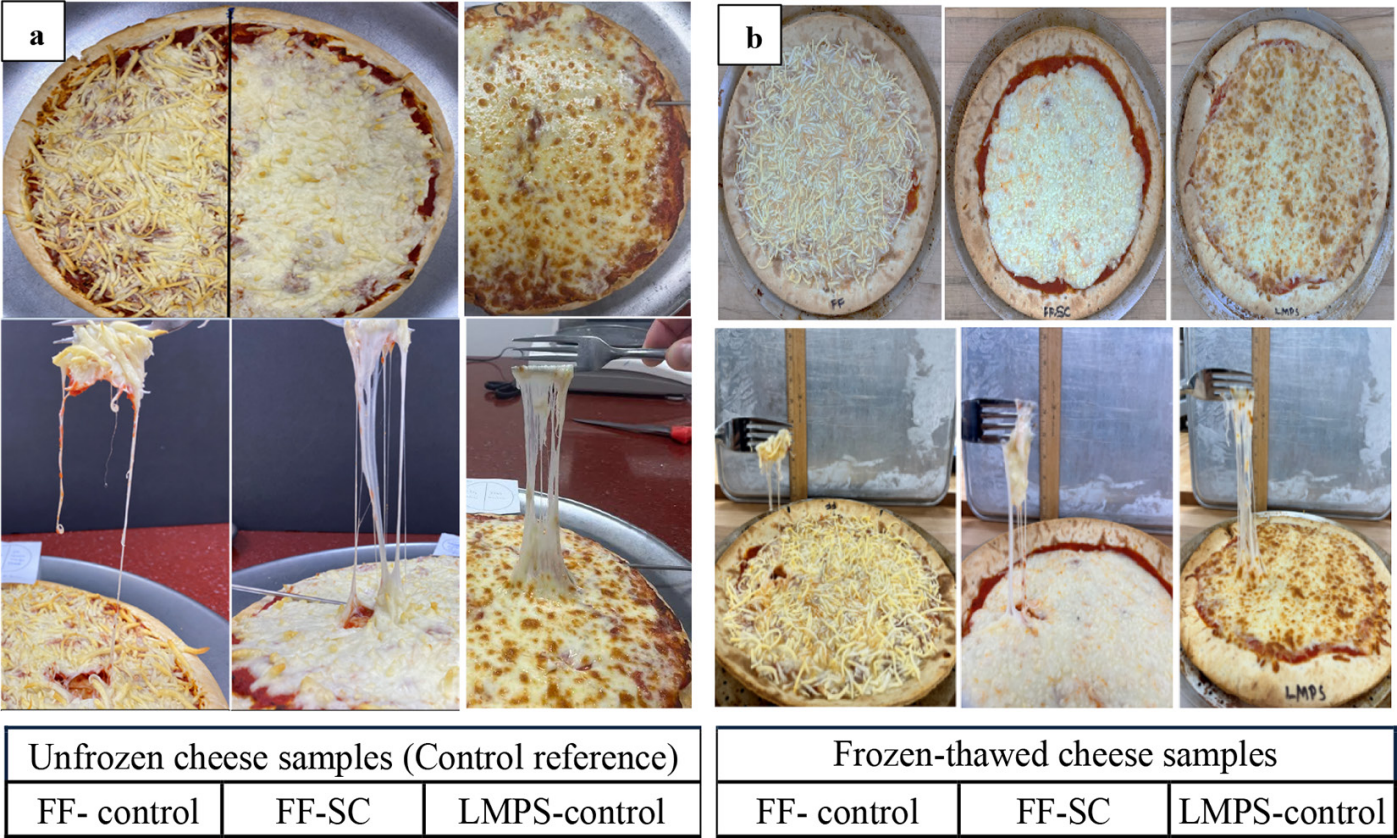
Pizza bake comparisons of unfrozen cheese samples control reference (a) and frozen-thawed cheese samples (b): comparisons of FF-control (fat-free cheese), FF-SC (fat-free soaked cheese), and LMPS-control (low-moisture part-skim mozzarella cheese) melt characteristics, shred identity, browning, and stretch by fork test. (Control reference images from our previous study, Sutariya et al., 2022).

Considering the large sizes of pizzas (12, 14, 16, and 18 inches), it is common for leftover slices to be reheated in a microwave before consumption. To replicate this scenario, previously baked pizza slices with FF-control, FF-SC, and LMPS-control cheeses were refrigerated for ∼4 h and then reheated in a microwave for 30 s. The shred identity and browning of the cheeses on reheated slices (Figure 2b) remained largely unchanged from their original baked state (Figure 2a). However, in terms of melting and stretching, the FF-SC sample showed enhanced melting and fewer strands, with the LMPS-control exhibiting slightly more melting along the slice edges and reduced strand formation (Figure 2a, b, and c). This increased melting and reduced strand formation in FF-SC and LMPS-control samples may be attributable to steam generation within the cheese matrix during microwaving. Steam builds internal pressure, leading to expansion and softening of the matrix (Gulzar et al., 2020). Alternatively, these changes could be due to overheating during the 30-s microwave cycle. Regardless, the steam or overheating effects were insufficient to improve the meltability of the FF-control cheese, likely due to its excessively dry and hardened surface.

**Figure 2:**
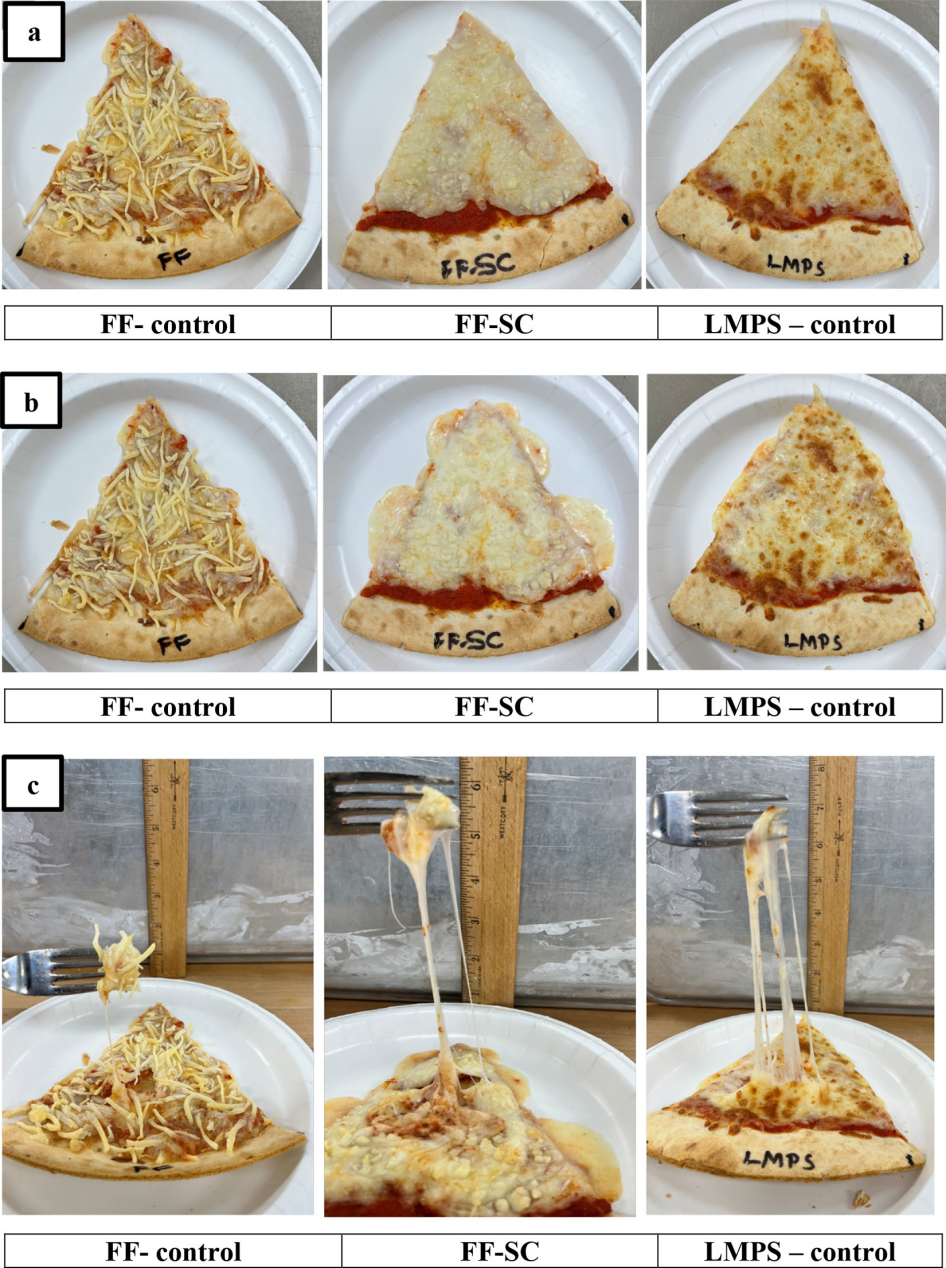
Pizza reheat comparisons of FF-control (fat-free cheese), FF-SC (fat-free soaked cheese), and LMPS-control, where pizza was first baked and cooled for 4 h, and pizza slices were then reheated in a microwave oven to study the effects of reheating a pizza slice, to mimic the leftover pizza consumption experience. (a) Appearance: first baked and cooled (4 h) pizza slice, evaluation of melt characteristics, shred identity, and browning. (b) Appearance: pizza slice after reheating in microwave oven for 30 s, evaluation of melt characteristics, shred identity, and browning. (c) Cheese stretch: pizza slice after reheating in microwave oven for 30 s, evaluation of stretch by fork test.

Meltability is a critical functional attribute of cheese, particularly in hot dishes such as burgers, lasagna, fondue, raclette, tartiflette, truffade, rebluchonnade, aligot, grilled cheese, pasta, and most notably, pizza (Gunasekaran and Ak, 2002; Wang and Sun, 2002; Altan et al., 2005). Meltability refers to how easily and to what extent cheese flows when heated. This property is especially important for mozzarella on pizza, where its ability to melt and stretch significantly enhances the eating experience. When mozzarella is heated on a pizza, it changes from a solid to a thick, flowing texture, an essential shift that creates the ideal melt and stretch. Rheological testing using a rheometer can quantify this behavior by measuring Gʹ and G″ as temperature increases. The point at which the cheese begins to flow, known as the sol-gel transition or melting point, is identified when the ratio Tan δ (G″/Gʹ) exceeds 1. A Tan δ value below 1 indicates solid-like behavior, and a value above 1 reflects viscous behavior. Due to differences in composition such as protein, fat, and moisture content, FF-control, LMPS-control, and FF-SC samples are expected to show variations in Gʹ, G″, and melting point. The FF-control is anticipated to have a higher Gʹ and a higher melting temperature (Tan δ >1) compared with LMPS-control. This study aimed to determine whether water addition in FF-SC cheese (69.7% moisture) could adjust its Gʹ and melting point to more closely resemble LMPS-control, improving its meltability. By analyzing rheological parameters (Gʹ, G″, and Tan δ) across a temperature ramp (30–70–30°C), we can better understand changes in texture such as initial firmness, melting behavior, and post-cooling hardness across the 3 mozzarella types.

To assess the differences in solid-like behavior at 30°C before heating among LMPS-control, FF-control, and FF-SC cheese samples, their storage modulus (Gʹ) values were compared. As expected based on the physical firmness of the cheese shreds, FF-control exhibited the highest Gʹ value at 30°C (Figure 3a), indicating the greatest rigidity. In contrast, FF-SC showed the lowest Gʹ value (Figure 3c), suggesting a softer texture. These findings align with previous studies (Karimi et al., 2015; Rathod et al., 2025), which also reported higher Gʹ values in fat-free mozzarella compared with fat-containing variants such as LMPS. The elevated Gʹ in FF-control can be attributed to its higher protein concentration (∼32.6%) and minimal fat content (<0.5%) compared with LMPS-control (∼21.3% protein, ∼21% fat) and FF-SC (∼21.6% protein, < 0.3% fat). The increased protein content promotes stronger protein-protein interactions, contributing to its firmness. Additionally, the low-fat content in FF-control has little effect on disrupting these interactions (Van Hekken et al., 2007).

**Figure 3:**
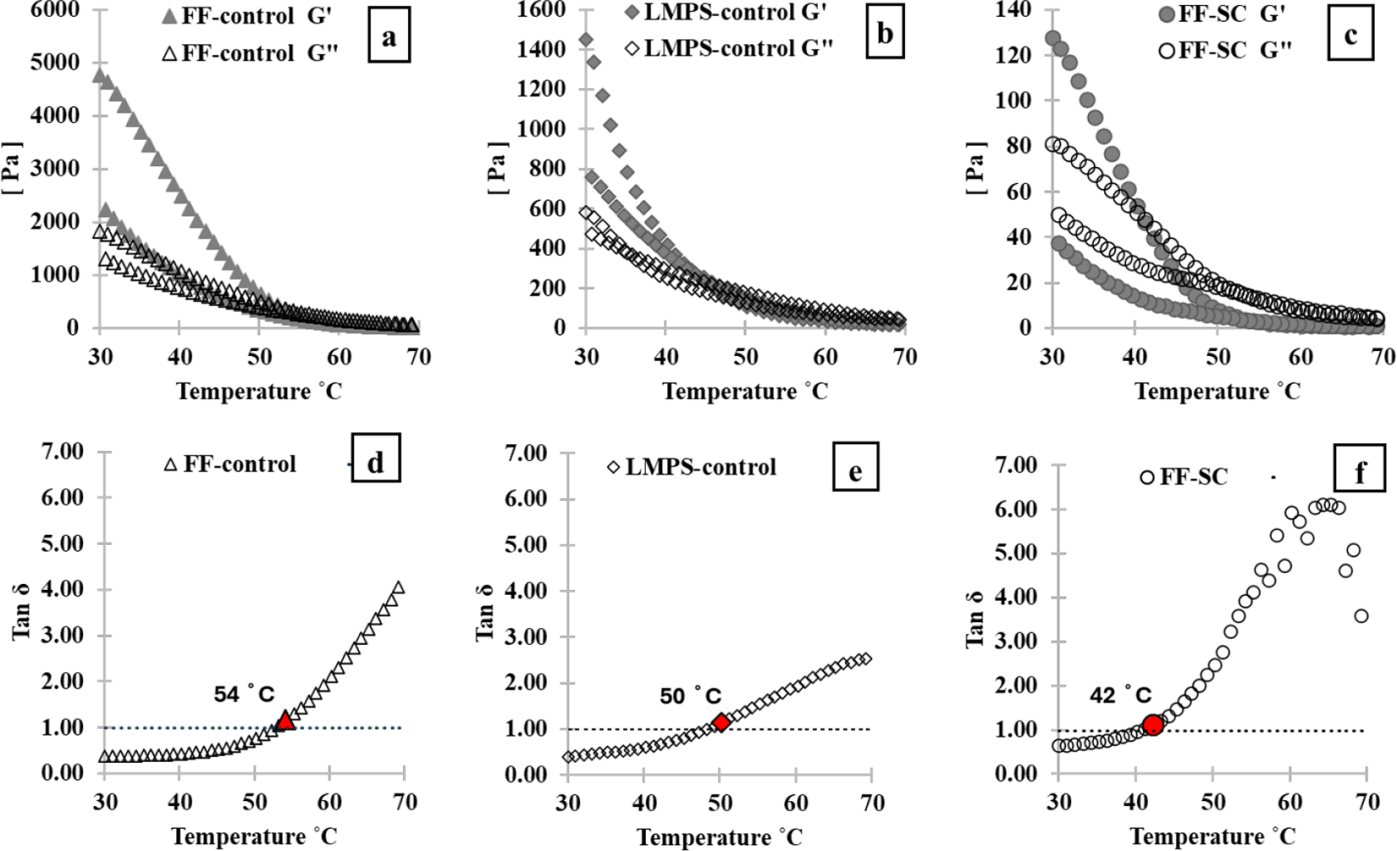
Temperature sweeps (a, b, c) and cheese melting temperatures (d, e, f). Temperature sweep from 30°C–70°C–30°C at constant 0.5% shear strain and 1-Hz frequency, with 1°C temperature increase at every 20 s: (a) FF-control G′ (▴), G″ (△); (b) LMPS-control G′ (♦), G″ (◊); and (c) FF-SC: G′ (•), G″ (○). Cheese melting temperature determined by Tan δ >1 values; temperature sweep from 30°C–70°C at constant 0.5% shear strain and 1-Hz frequency, with 1°C temperature increase at every 20 s: (d) FF-control Tan δ (△); (e) LMPS-control Tan δ (◊); and (f) FF-SC Tan δ (○). Error bars for SE of mean (n = 3) not displayed, for better visual clarity of graphs.

Melting temperature, determined by the point at which Tan δ (G″/Gʹ) exceeds 1, revealed that FF-control had the highest melting point (54 ± 0.04°C), followed by LMPS-control (50 ± 0.02°C), and FF-SC with the lowest (42 ± 0.12°C; Figure 3d, e, and f). As cheese is heated from 30 to 70°C, it undergoes structural changes influenced by its fat, protein, and moisture content. Fat begins to melt between 33 and 37°C, separating from the protein matrix, with increase in temperature (from 30 to 70°C) making cheese flowable due to weakening of the protein network rigidity (Wetton et al., 1991; Prentice et al., 1993; Dealy and Wissbrun, 2012). These combined effects determine the cheese's rheological behavior and meltability (Karlsson and Hauert, 2008). The higher melting point of FF-control compared with LMPS-control is primarily due to its low fat content, as fat melts at lower temperatures than protein. In LMPS cheese, the presence of fat globules disrupts protein-protein interactions and acts as plasticizer within the matrix, reducing the energy required for melting. As a result, LMPS melts more easily and flows better when heated (Paulson et al., 1998). In fat-free mozzarella cheeses (both FF-control and FF-SC), the absence of fat means that their melting behavior is primarily governed by protein and moisture content. Although proteins do not melt in the traditional sense, the softening of CN and certain serum-phase proteins between 30°C and 70°C contributes to the melting process (Paulson et al., 1998; Lucey et al., 2003). At elevated temperatures, the melting characteristics of cheese are influenced by the strength and number of CN-CN interactions, which are maintained through hydrogen bonding, hydrophobic forces, calcium cross-linking, and electrostatic interactions. As temperature rises, these bonds generally weaken, leading to a softer CN matrix (Park et al., 1984). Although hydrophobic interactions tend to strengthen with heat, the overall gel structure may still weaken due to reduced contact between CN molecules (Zoon, 1988). Additionally, increased electrostatic repulsion and decreased hydrogen bonding further destabilize the matrix, shifting the balance toward weaker protein-protein interactions and promoting softening and melting (Bryant and McClements, 1998; Lucey et al., 2003). In the FF-SC sample, the greater spacing between CN micelles resulting from its lower protein-to-moisture ratio of 0.30 (∼21.6% protein, ∼69.7% moisture), compared with 0.44 for LMPS-control (∼21.3% protein, ∼48.5% moisture) and 0.60 for FF-control (∼32.6% protein, ∼54.4% moisture), leads to reduced protein-protein interactions. Additionally, soaking cheese in water may lead to some calcium being drawn out from the CN matrix into the water, which can weaken the protein structure. These factors contribute to the lower melting temperature of FF-SC (Figure 3d, e, f) and lower Gʹ values (Figure 3a, b, c) throughout the heating range of 30 to 70°C, following the order FF-SC < LMPS-control < FF-control.

To evaluate cheese behavior during the cooling phase (70°C to 30°C; Figure 3a, b, c), Gʹ and G″ values were compared across the 3 samples. All cheeses showed increase in Gʹ and G″ during cooling and attempted to recover their original values at 30°C. However, none fully regained their initial firmness. Both LMPS-control and FF-control exhibited higher Gʹ than G″ upon resolidification, indicating a return to solid-like behavior (Figure 3a and b). In contrast, FF-SC showed lower Gʹ than G″, suggesting viscous behavior after cooling (Figure 3c). This viscous behavior in FF-SC may be due to its low protein concentration (∼31%), which limits the reformation of strong protein-protein interactions. Additionally, the soaking process may disrupt the balance between soluble and insoluble calcium, causing calcium ions to migrate from CN micelles to the serum phase and leading to protein leaching (Luo et al., 2013). These combined effects reduced protein interactions, and calcium-induced protein depletion likely weakened the structural integrity of the casein matrix, contributing to the viscous nature of the resolidified FF-SC cheese.

The method of soaking FF-SC in a pH-adjusted salt solution noticeably improved their baking properties, making them comparable to LMPS cheese. Compared with FF-control, soaked fat-free mozzarella (FF-SC) showed improved melting, stretchability, and browning during pizza baking, with benefits persisting after freeze-thaw cycles and microwave reheating. Rheological analysis confirmed lower melting temperature and more flowable texture. Soaking also enables faster thawing and shorter bake times, though flavor matching to LMPS-control remains a challenge. This approach enhances fat-free mozzarella's functional properties for pizza.
